# Implementation of data-cube pump–probe KPFM on organic solar cells

**DOI:** 10.3762/bjnano.11.24

**Published:** 2020-02-12

**Authors:** Benjamin Grévin, Olivier Bardagot, Renaud Demadrille

**Affiliations:** 1 Univ. Grenoble Alpes, CNRS, CEA, IRIG-SyMMES, 38000 Grenoble, France

**Keywords:** bulk heterojunctions, Kelvin probe force microscopy (KPFM), organic photovoltaics, photocarrier dynamics, pump–probe configuration, time-resolved measurements

## Abstract

An implementation of pump–probe Kelvin probe force microscopy (pp-KPFM) is reported that enables recording the time-resolved surface potential in single-point mode or over a 2D grid. The spectroscopic data are acquired in open *z*-loop configuration, which simplifies the pp-KPFM operation. The validity of the implementation is probed by measurements using electrical pumping. The dynamical photoresponse of a bulk heterojunction solar cell based on PTB7 and PC_71_BM is subsequently investigated by recording point-spectroscopy curves as a function of the optical power at the cathode and by mapping 2D time-resolved images of the surface photovoltage of the bare organic active layer.

## Introduction

Many emerging photovoltaic technologies rely on the use of thin film materials displaying structural and/or chemical heterogeneities at the μm or nm scale. This is the case for solution-processed organic donor–acceptor blends called bulk heterojunctions (BHJ), for polycrystalline direct bandgap semiconductors such as CdTe, CuIn*_x_*Ga_(1−_*_x_*_)_Se_2_ and Cu_2_ZnSnS_4_ and for hybrid organic–inorganic perovskite solar cells. Whatever material used, improving the performance of the solar cell requires a precise understanding of the relationship of the structural, chemical and optoelectronic properties of the device. Especially, a universal problem in third-generation photovoltaics consists in identifying the sources of carrier loss by the recombination of photogenerated charge carriers.

This has prompted the development of new time-resolved extensions of electrostatic force microscopy (EFM) and Kelvin probe force microscopy (KPFM). Time-resolved EFM (trEFM) has been used to map photoinduced charging rates (i.e., the time needed to reach an electrostatic equilibrium after illumination) in organic donor–acceptor blends with sub-ms time resolution [1]. Subsequent works have shown that sub-μs time resolution can be achieved by acquiring the full information on the cantilever oscillation, leading to the development of fast trEFM [2] and general-mode KPFM [3].

Contrary to feedback-free electrostatic methods [4–5], conventional KPFM relies on a closed feedback loop that compensates the tip–sample contact potential difference (CPD). It is thus inherently a rather “slow technique”. Kelvin controllers typically operate with time constants of a few to tens of ms. To implement time-resolved KPFM, a first method consists in increasing the detection bandwidth by using the so-called heterodyne mode [6–9] or dissipative electrostatic force modulation [10–11].

To probe the photocarrier lifetime in photovoltaic materials, another option consists in analyzing the dependence of the time-averaged KPFM compensation potential (or surface potential (SP)) on the frequency modulation (*f*_mod_) of an illumination source. The first demonstration of light intensity-modulated KPFM (IM-KPFM) was carried out in 2008 by Takihara et al. on polycrystalline Si solar cells [12], and it has been recently applied to organic [13–14] and nanocrystal-based [15] solar cells. However, there are some disadvantages to using this technique. First, at specific frequencies, the excitation signal used to generate charge carriers can interfere [16] with the cantilever oscillation or the ac voltage applied for the detection of the CPD. Errors in the surface photovoltage (SPV) measurement caused by photoinduced changes in the capacitance gradient [17] can also be a problem. Upon modulated illumination, the KPFM loop indeed measures the time-averaged value of the instantaneous SP weighted by the capacitance gradient [17] instead of the time-averaged SP. This may lead to a frequency-dependent overestimation of the average SP and consequently generate errors in the mathematical fit performed on the SP(*f*_mod_) curves, which is done to calculate the SPV decay-time constants. Last, the analysis of IM-KPFM data becomes a complex matter when the photocharging time is not negligible compared to the light pulse duration. In this case, numerical simulations are necessary to properly analyze the spectroscopic SP(*f*_mod_) curves [18].

When investigating organic donor–acceptor (D–A) blends, both capacitive effects and photocharging dynamics shall be taken into account, which renders the interpretation of IM-KPFM data even more difficult. Upon illumination, the capacitive junction formed by the cantilever tip and the conducting substrate onto which the organic layer is deposited is indeed reduced due to photogenerated carriers [19]. As a first approximation, this effect can be understood by assuming that there are no permanent charges in the “dark” (i.e., unilluminated) state of the organic layer considered as an undoped semiconductor. In “real” samples trapped carriers and electrostatic dipoles at the donor–acceptor interfaces contribute to the global electrostatic landscape probed by KPFM in the dark state [20]. The photocharging dynamics can be understood as follows. After exciton splitting and dissociation of the charge transfer states at the D–A interfaces, the photogenerated carriers experience a drift-diffusion limited by the carrier mobility [21]. Internal electric fields due to band bending at the D–A interfaces and at the organic/substrate interface play a key role in this process. In an open-circuit configuration occurring in KPFM experiments, the charges will move until the internal electric fields are compensated. Then the charge motion stops and the charge recombination balances the photogeneration. At this stage, the electrostatic landscape probed by KPFM reaches an equilibrium state.

**Figure 1:**
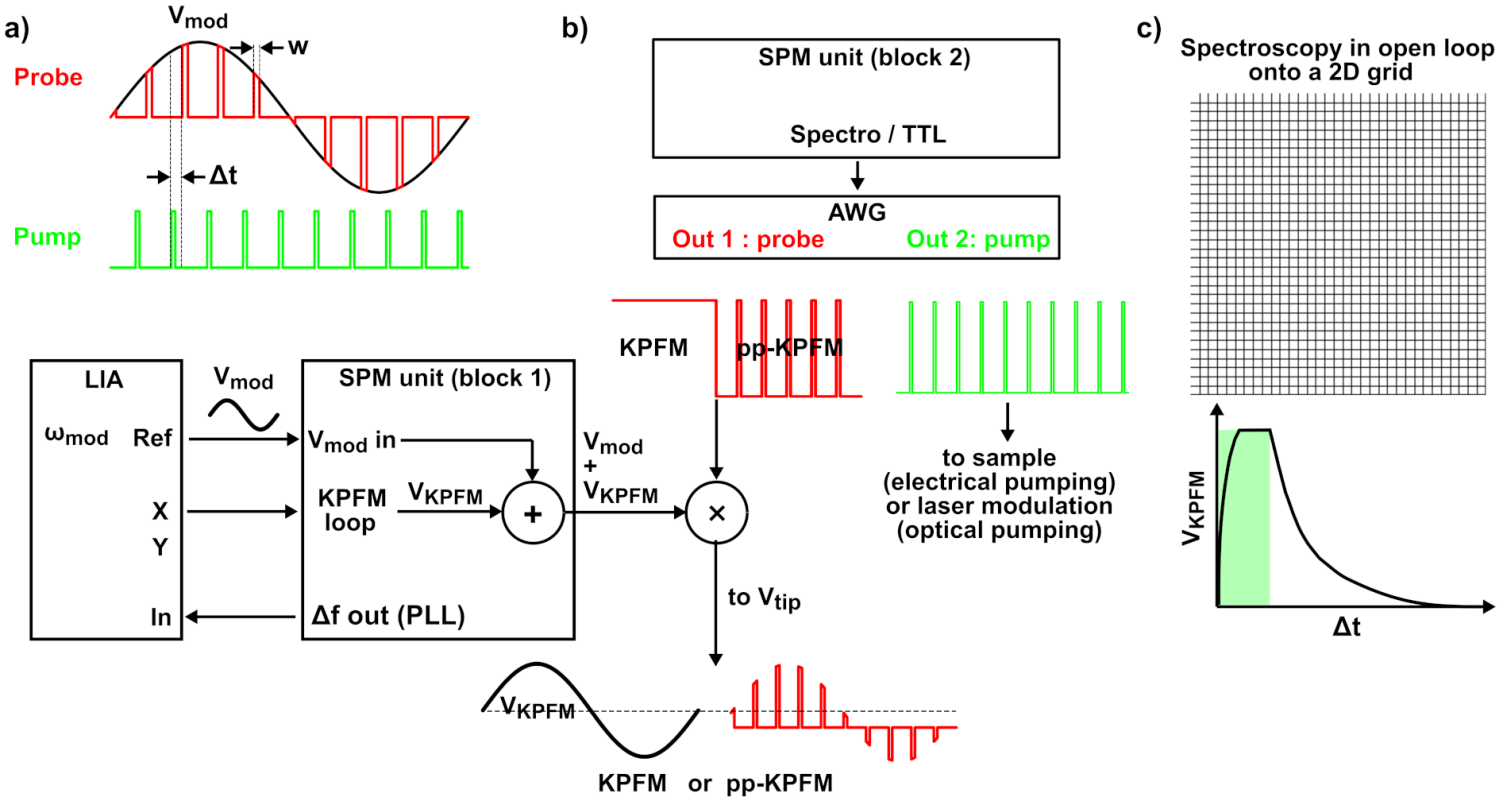
(a) Principle of pump–probe KPFM (pp-KPFM). In conventional KPFM, a sinusoidal ac voltage *V*_mod_ of frequency ω_mod_ is applied to the tip, and the electrostatic forces are detected with a lock-in amplifier (LIA). In pp-KPFM, the tip voltage consists of pulses with the same sinusoidal envelope of ω_mod_, which is used by the LIA as the reference for the detection of the electrostatic forces. Consequently, the KPFM loop compensates only the tip–sample contact potential difference (CPD) that exists during the pulse of width *w*. The time-evolution of the CPD is monitored by recording the voltage at the output of the KPFM loop (*V*_KPFM_) as a function of the delay Δ*t* between the probe and pump pulses. (b) Scheme (top middle and bottom left-middle) of the signal processing by the scanning probe microscope (SPM) controller, the LIA and the arbitrary waveform generator (AWG). The electrostatic forces are detected by feeding the frequency-shift signal (Δ*f*) from the SPM phase-lock loop to the LIA input (KPFM operated in frequency-modulation mode). Using the SPM unit (block 1), the reference bias modulation voltage (*V*_mod_, ω_mod_) is added to the compensation voltage generated by the KPFM feedback loop. The sum is multiplied by the output of the first channel of the AWG dedicated to the probe signal, which delivers a continuous voltage level or continuous voltage pulses. The setup can be automatically switched from standard KPFM to pp-KPFM configuration. The second AWG channel generates the pulses that are used for electrical pumping of the sample or for optical pumping by digitally modulating a laser unit, which is used for sample illumination. Transistor–transistor logic (TTL) signals synchronized with the spectroscopic ramps are generated by the SPM unit to trigger the generation of sequences of probe and pump pulses with predefined delays. (c) Spectroscopic curves of the KPFM potential *V*_KPFM_ as a function of the pump–probe delay time Δ*t* can be acquired at selected locations or on a predefined 2D grid (data-cube mode).

Pump–probe Kelvin probe force microscopy (pp-KPFM) is a promising alternative to IM-KPFM. It is a priori not prone to capacitive artefacts, and it offers the possibility to probe directly and independently both the photocharging rate and the SPV decay. As introduced by Murawski et al. [22], in pp-KPFM the modulated bias voltage, which is used for the detection of electrostatic forces with a lock-in amplifier (LIA), is restricted to a finite time window (*w*, Figure 1a). Consequently, the counter potential generated by the KPFM loop compensates only the CPD that exists during this time window. By recording the KPFM signal as a function of the delay time Δ*t* between the time window during which the ac modulation is applied (i.e., the probe) and the signal used to generate SP transients (the pump), one can track the SP evolution as a function of time.

The pump can be either an electrical (voltage pulse) or an optical (light pulse) signal. The first configuration was used by Murawski et al. to demonstrate the ability of pp-KPFM to probe the charge dynamics in organic field effect transistors (OFETs) at a µs temporal resolution [23]. For that purpose, they recorded successive images of the SP in the active channel of an OFET device, each of them being acquired at a different delay between the probe and a voltage pulse applied to the drain electrode of the transistor. In a later study, Schumacher et al. [24] used optical pumping in a pp-KPFM approach to probe the photocarrier lifetime in GaAs samples grown at low temperature.

In organic BHJ solar cells, electron donor and acceptor materials are processed to form two interpenetrated networks phase-segregated at the 10 nm scale. Consequently, if one aims at investigating the interplay of the morphology and the photocarrier dynamics in BHJs (and by extension in other nanostructured photovoltaic materials), it is necessary to probe SP transients upon pulsed illumination at a high spatial resolution. Similar to the study of OFETs [23], a first option may consist in recording successive sequences of pp-KPFM images of the same sample area using a variable pump–probe delay time. However, at room temperature, as usually defined for studies of solar cells, it is hardly possible to record a stack of images while keeping exactly the same tip–sample relative positioning because of thermal drift and piezo creep. To avoid lateral misalignment artefacts, a better option is to record a matrix of spectroscopic curves of the pp-KPFM signal on a 2D grid. In this work, we explore the performance of such a spectroscopic pp-KPFM approach in data-cube mode. We show how topographic artefacts can be avoided by implementing an automated sequence that allows for performing the scan with a standard KPFM configuration and acquiring the spectroscopic pp-KPFM curves with an open *z*-loop. The implementation is validated by carrying out test measurements by electrical pumping of reference substrates. Moreover, the technique is applied to characterize BHJ solar cells by optical pumping. To this end, point-spectroscopy curves are recorded on the device cathode as a function of the optical power using various values of the pump–probe time delay. Furthermore, the mapping of 2D time-resolved images of the bare active organic layer is employed. The results demonstrate that the SPV dynamics are dominated by trap-delayed processes in the investigated system. Current limitations and upcoming improvements of the chosen approach for pp-KPFM are finally discussed.

## pp-KPFM Implementation

The experiments were performed on the basis of noncontact AFM (nc-AFM) under ultrahigh vacuum (UHV) with a beam deflection setup operated in frequency-modulation (FM) mode at room temperature. In the following, we only describe the general setup that has been used to implement data-cube pump–probe-KPFM (Figure 1b and Figure 1c). Additional technical information is provided in the experimental section.

We kept the standard SPM controller configuration for frequency-modulation KPFM (FM-KPFM). Here, the electrostatic forces are detected by demodulating the modulated component (ω_mod_) of the frequency-shift signal (Δ*f*) with the LIA. The reference bias modulation voltage (*V*_mod_, ω_mod_) and the compensation voltage generated by the KPFM feedback loop (*V*_KPFM_) are internally summed by the SPM unit.

To generate the modulated bias for pp-KPFM, a pseudo multiplication is performed on this voltage sum by using a fast analog switch, the TTL input of which is driven by one of the two outputs of a programmable AWG. In pp-KPFM, the CPD is only detected during the time window defined by the probe pulses. Murawski et al. have shown that this can generate artefacts in the *z*-regulation [22], since for any given pump–probe delay, the time-averaged CPD differs from the one probed and compensated by the KPFM loop. In our case, a similar issue occurs since the compensation bias is only applied during the probe-time window, keeping in mind that the multiplication by the pump train pulses is applied to the sum of *V*_mod_ and *V*_KPFM_. As a result, the electrostatic forces are not compensated during the time interval between the probe pulses, and the *z*-feedback can be affected by the time-variable electrostatic force field. To minimize topographic artefacts, a first option may consist in using a dual set of KPFM compensation loops operated at different modulation frequencies [22]. The first loop would be used for pp-KPFM and the second to compensate the time-averaged component of the electrostatic potential.

**Figure 2:**
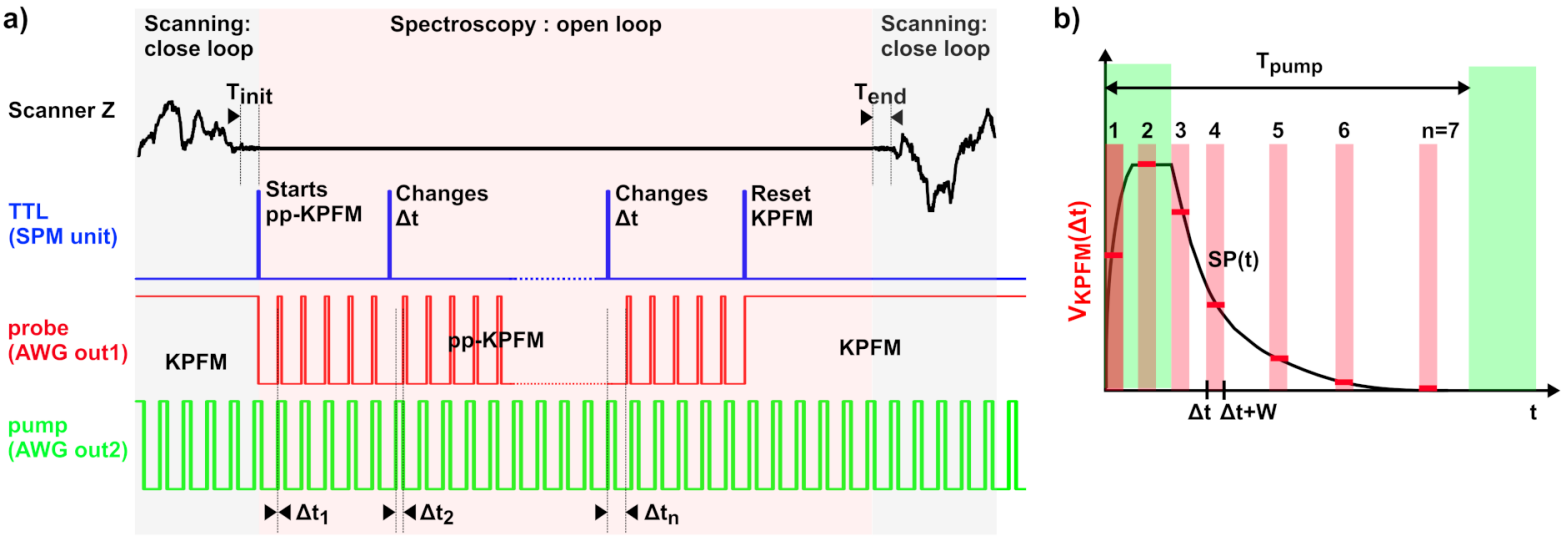
(a) Detail of the spectroscopic sequence implemented for the acquisition of the pp-KPFM signal as a function of the pump–probe delay Δ*t*. The topographic scan is performed by closed-loop *z*-regulation simultaneously with “conventional” FM-KPFM imaging. Before recording each spectroscopic pixel, the tip is stopped. The *z*-regulation is kept during an initial stabilization delay prior to spectroscopy (*T*_init_), and then the *z*-loop is frozen during the spectroscopic ramp. The KPFM configuration is changed to pp-KPFM by the first TTL pulse, and each subsequent TTL pulse changes the pump–probe delay. The last TTL pulse resets the setup configuration in conventional KPFM. The *z*-regulation is reactivated, and nc-AFM/KPFM scanning resumes after a final stabilization delay (*T*_end_). (b) Illustration of a spectroscopic curve acquired in the case of optical pumping. The instantaneous surface potential SP(*t*) is symbolized by the black line. *V*_KPFM_(Δ*t*) curves are acquired for a predefined number of discrete pump–probe delays. Note that the Δ*t* values can be either evenly or irregularly distributed. The time-resolution is fixed by the width (*w*) of the probe pulses. The horizontal red bars represent the pp-KPFM potential. It is equal to the time-integral value of SP(*t*) normalized by *w* over the interval Δ*t* − Δ*t* + *w*.

In this work, we propose an alternative method that consists in operating the KPFM in standard mode (closed-loop *z*-regulation and sinusoidal bias modulation) for the topographic analysis and in switching the setup configuration to pp-KPFM with an open *z*-loop for the spectroscopic acquisition of *V*_KPFM_(Δ*t*) curves (Figure 1b and Figure 2). Great care was taken to stabilize the setup before spectroscopic acquisition in open-loop configuration in order to minimize the impact of the *z*-drift on the KPFM potential. The residual *z*-drift less was smaller than 0.4 nm over a time lapse of 40 s, see Figure S1 in Supporting Information File 1. Switching the controller configuration from standard KPFM to pp-KPFM was done by synchronizing the AWG unit with the spectroscopic ramps of the SPM controller by means of TTL pulses and by using predefined sequences of pulses and continuous-wave (cw) dc signals stored in the memory of the AWG. Note that in this configuration, the KPFM potential probed during the topographic acquisition yields a time-averaged value of the SP since the pump signal is permanently applied to the sample. A further refinement, not shown in Figure 2, consists in switching also the pump signal to a cw level during the topographic acquisition, basically given by an “on” or “off” state. This can be done for the purpose of specific tests described hereafter. However, for optical pumping, it is preferable to keep the pump permanently in a modulated configuration as shown in Figure 2 to maintain a continuous time-averaged optical power on the cantilever to avoid thermal detuning effects.

Finally, one must keep in mind that pp-KPFM does not exactly probe the instantaneous SP but its time-averaged value over the time window defined by the probe pulse. This point is illustrated in Figure 2b. Moreover, we underline that in our approach the series of discrete pump–probe delay values used for the spectroscopic acquisition can be distributed either uniformly or irregularly within the time window defined by the pump period (see Figure 2b). The second configuration can be used to reduce the spectroscopic acquisition time by using less data points to probe the parts of the time-domain where the SP evolves more slowly.

## Organic BHJ Solar Cells

**Figure 3:**
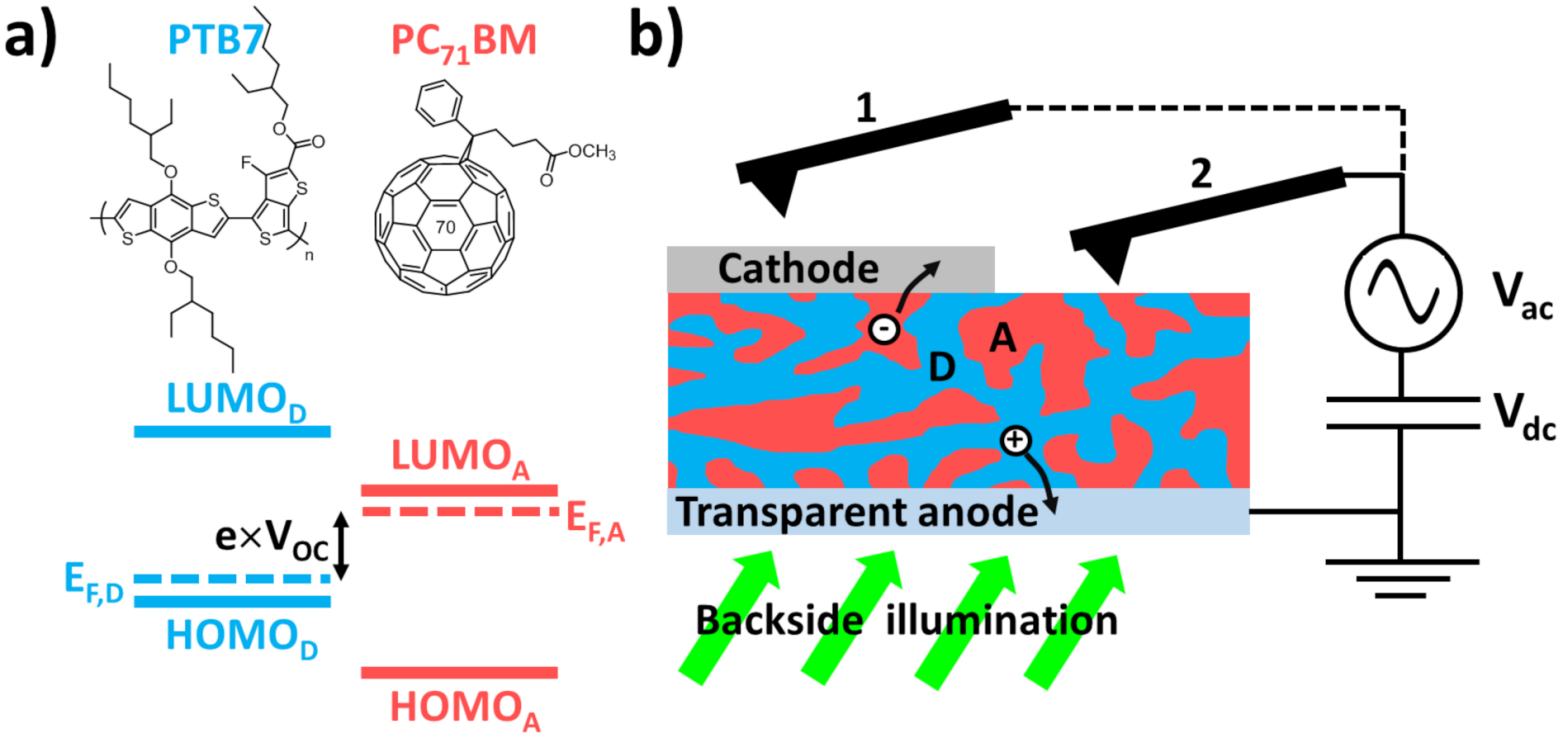
(a) Top: chemical structures of PTB7 (electron donor, D) and PC_71_BM (electron acceptor, A). Bottom: illustration of the type-II energy level alignment between the electron donor and the electron acceptor. The open-circuit voltage (*V*_OC_) is determined by the splitting of the quasi-Fermi levels of holes and electrons upon illumination, symbolized by dotted lines. (b) Organic BHJ solar cell and experimental configuration. The sample is illuminated in backside geometry. The transparent anode, the hole collecting electrode made of indium tin oxide coated with PEDOT:PSS, is grounded. The modulation and compensation bias are applied to the tip. The pp-KPFM measurements are performed either on top of the cathode (1: single-point spectroscopy) or on top of the bare organic layer (2: data-cube spectroscopy).

In this work, PTB7:PC_71_BM BHJ photovoltaic thin films have been used as test samples (Figure 3) for pp-KPFM experiments. In the following, a few concepts of organic photovoltaics are presented. For a comprehensive introduction to this field, the reader may refer to [25].

Solution-processed organic solar cells [25] rely on the combination of electron donor (D) and acceptor (A) π-conjugated polymers and/or molecules that display a type-II energy offset between their highest occupied molecular orbital (HOMO) and lowest unoccupied molecular orbital (LUMO) levels. These energetic offsets enable to dissociate singlet excitons into Coulomb-bound electron–hole pairs also called charge transfer states (CTs). These can either recombine in pairs at the D–A interfaces or split up into free charges. The latter can eventually reach the collection electrodes of the device. Here, the low-bandgap polymer PTB7 was used as the donor and the fullerene derivative PC_71_BM as the acceptor.

In the BHJ configuration [26], the D and A materials should form two interpenetrated networks phase-segregated at the 10 nm scale, to maximize the donor–acceptor interfacial area where the excitons are dissociated and to overcome the short-exciton diffusion length. The vast majority of solution-processed D–A blends actually display more complex morphologies. For instance, they can feature a three-component organization in two (relatively) “pure” phases (i.e., donor-enriched and acceptor-enriched sub-networks), and a third one where donor and acceptor molecules are finely intermixed at the sub-10 nm scale [27]. Further complexity is added by the fact that radically different morphologies can be obtained depending of the solvent and additives used for the film deposition from solution.

The use of PTB7:PC_71_BM is widely documented. It is now well established that films of good morphology, namely with a nanoscale phase separation, can be obtained from solutions containing 1,8-diiodooctane (DIO) as solvent additive [28–30]. Adding a small amount of DIO indeed prevents the formation of large PC_71_BM aggregates and favors the formation of an intermixed morphology at the scale of a few tens of nm during film casting and drying.

In BHJs, the photogenerated carriers recombine in a nongeminate manner by electron–hole annihilation at the D–A interfaces. Here, we do not discuss the losses by exciton relaxation or by pairwise recombination of the CT state. The free carriers can also be trapped in tail states [25] before recombining with free unpaired counter charges. This slower recombination process is called a trap-assisted or trap-delayed recombination.

## Results and Discussion

### pp-KPFM upon electrical pumping

The ability to perform time-resolved measurements using this pp-KPFM implementation was validated by a series of single-point spectroscopy measurements applying electrical pumping on a highly oriented pyrolytic graphite (HOPG) substrate. The sample was electrically connected to the AWG by mounting the HOPG substrate onto a sample holder designed with in situ electrical contacts. The pump signal generated by the AWG was transmitted through a coaxial cable (air side) connected to a twisted pair (vacuum side) through an intermediate UHV feedthrough.

**Figure 4:**
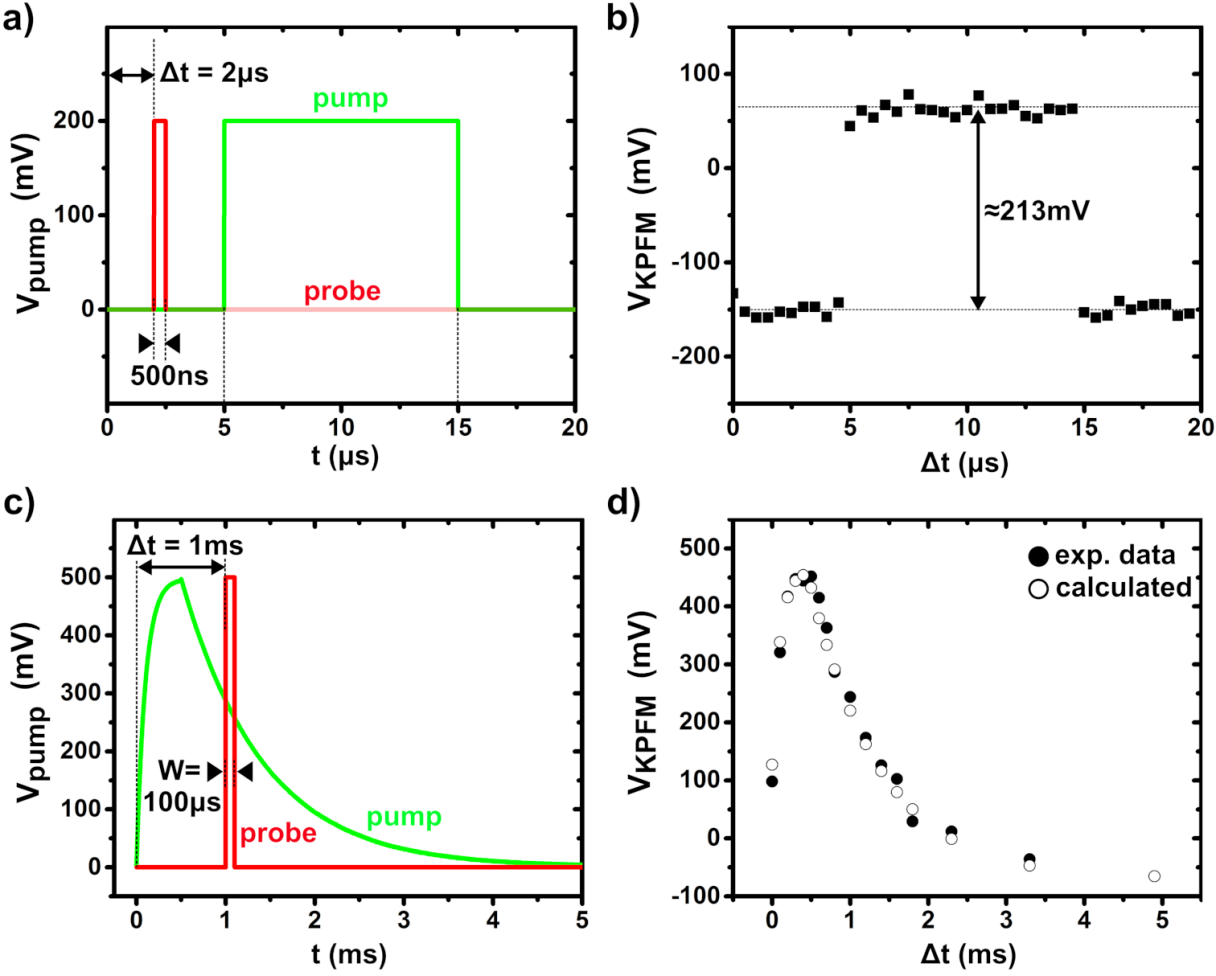
(a, b) pp-KPFM single-point spectroscopy of an electrical square-wave signal of 10 µs (pump, amplitude 200 mV, period 20 µs). (a) Plot of the pump and probe signals as a function of time for Δ*t* = 2 µs. The pump signal was recorded using the oscilloscope. NB: the *y*-scale is shown for the pump only. The probe-time window was set to 500 ns. (b) KPFM compensation potential as a function of Δ*t*. 40 delay values evenly distributed within the time window of 20 µs as defined by the pump signal period were used to record that spectrum. The integration time per pixel was 4 s. (c, d) pp-KPFM single-point spectroscopy measurement of a “surge pulse” of 5 ms (pump, amplitude 500 mV). (c) Plot of the pump and probe signals as a function of time for Δ*t* = 1 ms. The probe window was set to 100 µs. (d) KPFM compensation potential (average of 5 spectra) as a function of Δ*t* (filled circles). 17 delay values irregularly distributed within the time window of 5 ms were used. The integration time per pixel was set to 1 s, preceded by a pre-integration time of 2 s. The open circles represent calculated values obtained by integrating the pump signal over the probe-time window and by applying a correction factor to correct the impedance mismatch. The calculated data have been shifted along the *y*-axis to ease the comparison with the experimental results. This *y*-shift with respect to the zero baseline results from the difference of the tip–substrate work function.

Figure 4 presents the results of two measurements carried out with different pump and probe signals (note that two different cantilevers were used for these two tests). In the first example (Figure 4a and Figure 4b), a 50 kHz square-wave signal with a 200 mV amplitude and a 50% duty ratio was used for the pump. The probe width was set to 500 ns, and the pump–probe delay was incremented 40 times by steps of 500 ns during the spectroscopic acquisition. The pp-KPFM spectrum reproduces fairly well the shape of the pump signal, demonstrating that a time-resolution at least as good as 1 µs can be achieved with this setup. The slight overestimation of the pulse amplitude (213 mV instead of 200 mV) originates from an impedance mismatch effect. This has been confirmed by comparing the KPFM loop response (in standard mode) to the dc bias applied from the AWG or internally added to the tip by the SPM unit (see Figure S2 in Supporting Information File 1).

A pulse signal of 200 Hz and an amplitude of 500 mV featuring a sub-ms potential rise and a slower decay were used as the pump for the second test (Figure 4c and Figure 4d). Also here, an excellent agreement was obtained between the pump and pp-KPFM signals. More precisely, the pp-KPFM spectrum (filled circles in Figure 4d) matches the values calculated by integrating the pump signal over the time windows defined by the probe (open symbols in Figure 4d).

Here, we stress that to achieve a proper pp-KPFM measurement, it is crucial to set a suitable time constant for the KPFM feedback loop and an adequate integration time per pixel. The signal-to-noise (*S*/*N*) ratio dramatically decreases when switching from KPFM to pp-KPFM due to a reduction of the bandwidth [22]. This effect demands a reduction of the loop gain and an increase of the integration time. Namely, the KPFM loop needs sufficient time to adapt and track the SP changes that occur when changing the pump–probe delay. A pre-integration time during which the KPFM potential is not recorded shall precede the acquisition of each spectroscopic pixel. The pre-integration time can be set by repeating several times the same pump-probe delay in the probe’s pulse sequence (ex: Δ*t*_1_ = Δ*t*_2_ = Δ*t*_3_ in Figure 2a). This procedure was used to process the data shown in Figure 4d, and it has been systematically applied for pp-KPFM measurements using optical pumping.

### pp-KPFM upon optical pumping of the organic solar cell

The PTB7:PC_71_BM blend forming the active layer of the solar cell was processed from a chlorobenzene/DIO (CB/DIO) mixture (see Experimental section) with the aim to obtain an optimized nanoscale morphology [28]. The global performance deduced from macroscopic electrical characterization (Figure S3 in Supporting Information File 1) remains however below the state-of-the-art for PTB7:PC_71_BM-based devices. We will describe later that an imperfect morphology may be the origin of the reduced performance.

In a first step, the dynamic photoresponse of the device upon optical pumping was investigated by point spectroscopy at the cathode. This experimental configuration is labeled “1” in Figure 3b. It was chosen for an initial examination of the pp-KPFM operation using optical pumping, because the *S*/*N* ratio could be increased at will by averaging several successive spectroscopic curves. In this configuration, the cathode defines an equipotential level. In other words, the dynamic SP photoresponse is not position-dependent. Furthermore, here, the SPV can be directly compared to the open-circuit voltage deduced from the macroscopic electrical characterization.

**Figure 5:**
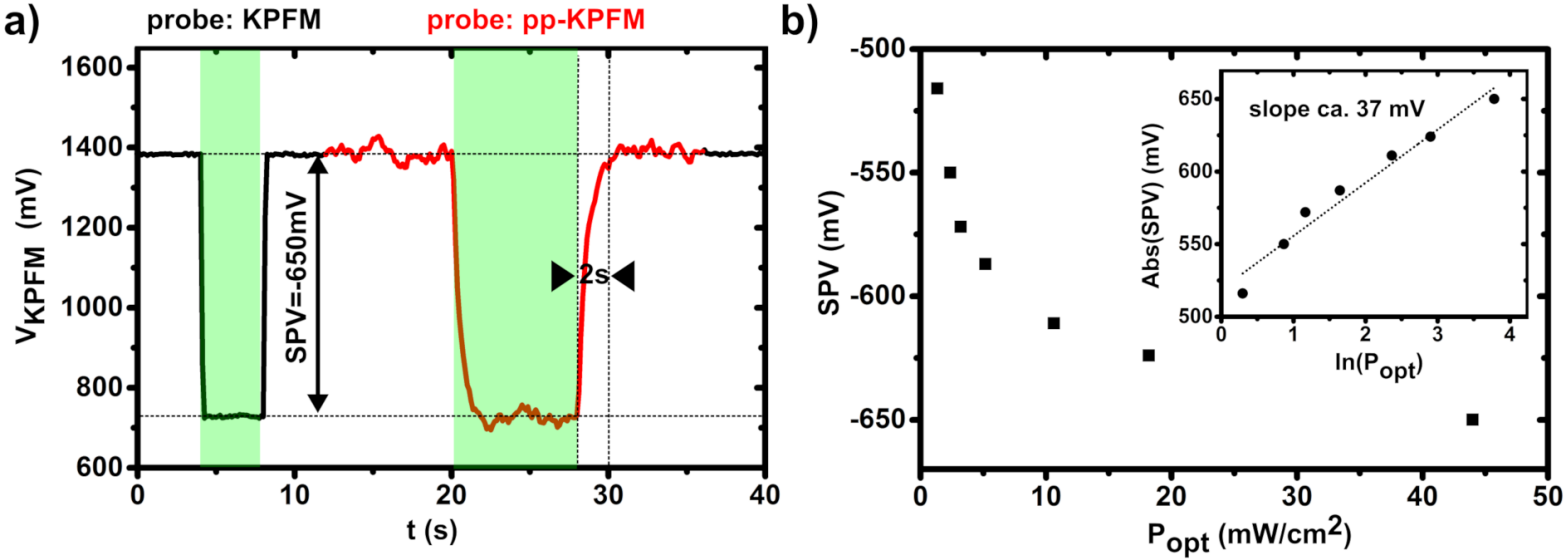
(a) Plot of the KPFM compensation potential measured at the solar cell cathode as a function of time (spectroscopic sequence of 320 pixels, 125 ms per pixel). The probe signal configuration is automatically switched from standard KPFM to pp-KPFM (pulses of 250 µs, repetition frequency of 200 Hz) at *t* = 12 s and set back to standard KPFM at *t* = 36 s. The illumination state is switched twice from dark to cw illumination. First illumination sequence: 4 s < *t* < 8 s, second sequence: 20 s < *t* < 28 s, *P*_opt_ = 48 mW∙cm^−2^ at 515 nm). (b) Plot of the surface photovoltage (SPV) as a function of the optical power *P*_opt_. The SPV is calculated as the difference between the KPFM potential values measured (standard KPFM configuration) at cw illumination and in the dark. Inset: plot of the SPV (in absolute value) as a function of the natural logarithm of the optical power.

Figure 5a shows a measurement of the SPV performed by applying a long cw light pulse (515 nm) to the sample. The calculated SPV of about 650 mV at 48 mW∙cm^−2^ and 515 nm is close to the open-circuit voltage of 680 mV upon one sun illumination as deduced from the electrical characterization. The SPV approximately displays a logarithmic dependence on the illumination intensity (Figure 5b), with a slope equal to ca. 1.5 *k*_B_*T*∙*q*^−1^ (*k*_B_: Boltzmann constant, *q*: electron charge). This suggests that trap-delayed processes constitute a significant pathway for photocarrier recombination in this sample [31]. These measurements were also performed to confirm the validity of pp-KPFM operations on this system. More precisely, we probed the ability of pp-KPFM to yield a proper SP measurement. For that purpose, the setup configuration was switched from standard KPFM to pp-KPFM mode within the spectroscopic ramp (Figure 5a), and two successive light pulses were applied. The first and the second light pulse occurred during KPFM and pp-KPFM, respectively. For clarity, we underline that there is no variable pump–probe delay. The pump channel is only used to apply dc levels corresponding to the on and off states of the illumination source. In other words, in this test, pp-KPFM is not used to perform a time-resolved measurement as a function of Δ*t*. It is rather used to monitor a SP at two different levels of illumination, namely in a dark state and under cw illumination. Neglecting noise, both configurations yielded identical SP levels in the dark and upon cw illumination. However, as expected, the noise level increases for pp-KPFM, and a much longer time delay is needed to stabilize the compensation potential upon turning on and off the illumination. As noted above, this urged us to use pre-integration delays when performing measurements of the pp-KPFM signal as a function of Δ*t*.

**Figure 6:**
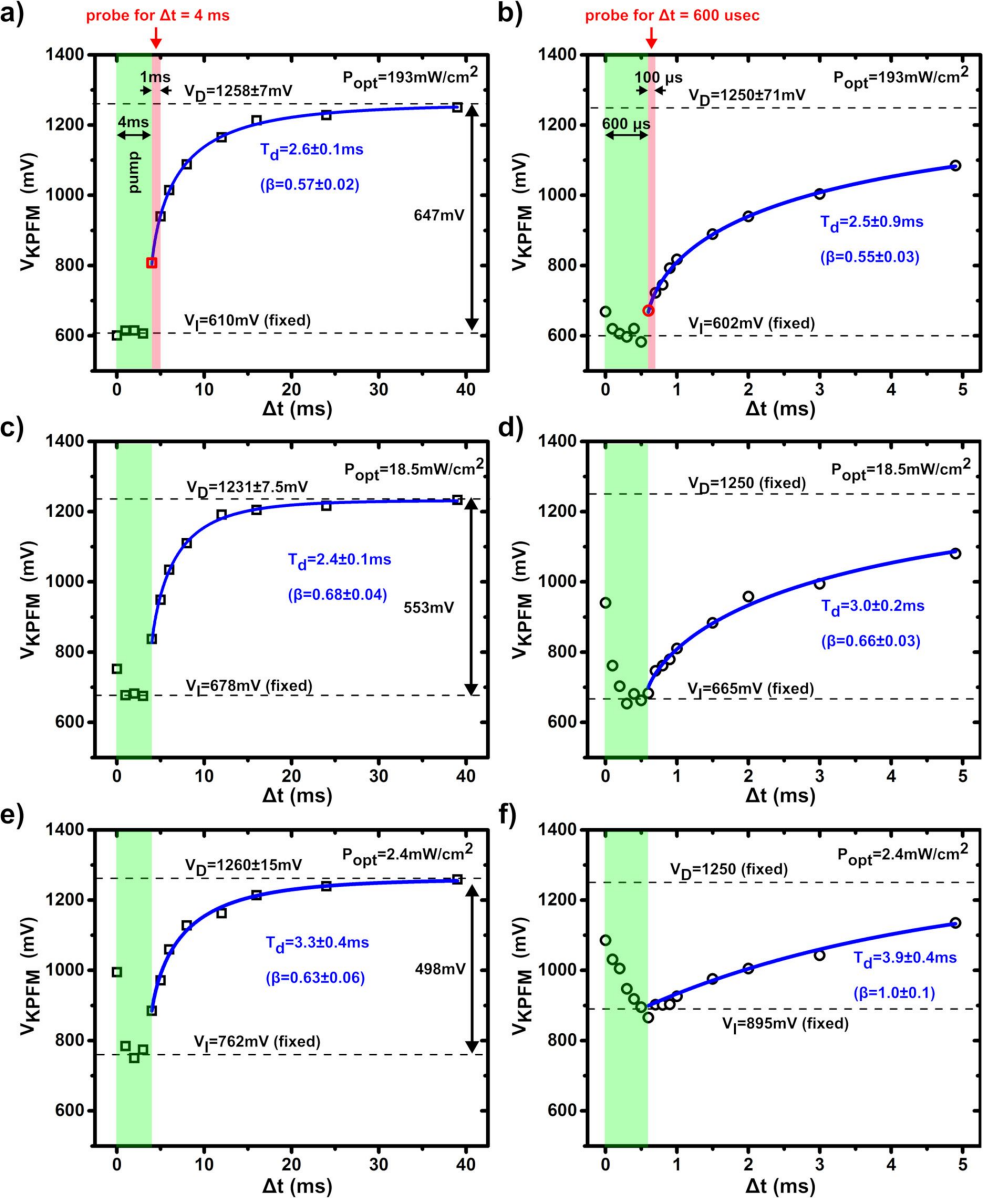
(a–f) Spectroscopic curves (average of 5 acquisitions) of the pp-KPFM compensation potential as a function of Δ*t* measured at the solar cell cathode. The data were acquired using three different illumination intensities (peak power of the pump pulses): (a, b)193 , (c, d) 18.5 and (e, f) 2.4 mW∙cm^−2^. Two different pump–probe delay sequences were used. Open squares and open circles represent data of the first and second sequence, respectively. (a, c, e) First sequence: 4 ms pump pulses repeated at 25 Hz, 1 ms probe-time window, post-data acquisition delay of 2 s, integration time 1 s. (b, d, f) Second sequence: 600 µs pump pulses repeated at 200 Hz, 100 µs probe-time window, post-data acquisition delay of 2 s, integration time 1 s. The time intervals corresponding to the pump (a–f) and the probe (a, b) signals (shown only for one given delay) are highlighted by half-transparent green and red rectangles, respectively. Note that the time (*t*) and delay (Δ*t*) scales coincide since the delays are defined with respect to the time origin *t* = 0 s. However, each data point represents a measurement of the SP integrated during the pump–time window. The solid blue lines represent the results of the numerical curve fits performed to extract the SPV decay-time constants (τ_d_). For the fit, the *V*_I_ values were fixed at the value reached upon illumination. *V*_D_, τ_d_ , β were free variables, with the exception of the data in (d) and (f), that were adjusted with fixed *V*_D_ values.

Time-resolved pp-KPFM measurements were carried out at the cathode (Figure 6) as a function of the illumination intensity and by using two different pump–probe delay sequences. The operating parameters are detailed in the caption of Figure 6. The experimental data were analyzed by modeling the SPV decay after light pulse extinction with a stretched exponential function (time constant: τ_d_, stretch exponent: β) and by integrating over the probe-time window (*w*). It can be shown that for given Δ*t*, the pp-KPFM potential in the “decay part” of the curve can be expressed as (see Supporting Information File 1):

[1]
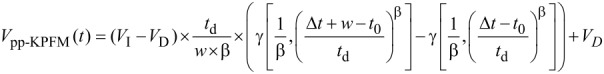


Here, *V*_D_ and *V*_I_ correspond to the dark-state SP and the SP value at the end of the light pulse (*t* = *t*_0_), respectively. If the pulse duration exceeds the photocharging time, *V*_I_ will be equal to the SP value measured upon cw illumination (*V*_cw_). γ represents the unnormalized lower-half Euler gamma function (see Supporting Information File 1).

Obviously, a trade-off between the time-resolution (limited by the probe-time window) and measurement of the full SPV dynamics must be found. In Figure 2a, a pump pulse of 40 ms is applied. Here, several tens of ms are needed to recover completely the dark-state SP after pulse extinction. Using a pump signal with a shorter period (5 ms instead of 40 ms, Figure 2b) increases the temporal resolution, but does not allow the system to return fully to its initial electrostatic state between the light pulses. However, it is important to note that the data acquired using these two different sequences display an excellent consistency. At the irradiance maximum (*P*_opt_ = 193 mW∙cm^−2^, Figure 6a and Figure 6b), the decay-time constants, stretch exponents and dark-state SP values extracted from both curves are identical within the error bars. The agreement between both data sets is unambiguous when plotting the two data sets in the same graph with a common normalized origin (Figure S5 in Supporting Information File 1).

As expected, the magnitude of the SPV decreases when reducing the optical power (Figure 6). However, the decay-time constant is barely fluence-dependent, which indicates that the underlying dynamics originate from trap-delayed processes [25]. Most likely, a broad distribution of states exists in which the photocarriers are trapped for longer or shorter periods as indicated by stretch exponents lower than 0.7 required for fitting of the spectroscopic curves (with the exception of the data in Figure 6f). These traps can be partially filled by applying a continuous white light background in addition to the pump pulse, which results in a significant reduction of the effective decay-time constant (see Figure S6 in Supporting Information File 1). However, these experimental conditions do not fulfil the requirements of a small perturbation measurement [32], and the SPV dynamics remain most likely dominated by trap-release processes.

Due to the limited time-resolution, the photocharging dynamics could not be addressed as accurately as the SPV decay dynamics. Aquiring all information at once would require using smaller probe-to-pump duty ratios. This could be done by keeping the same pump period while reducing the probe-time window. This was not possible in practice because of an insufficient *S*/*N* ratio. Some interesting observations can nevertheless be made. The photocharging time, i.e., the time needed to reach an electrostatic equilibrium upon illumination, appears to depend strongly on the pump fluence. More precisely, the photopotential builds up more slowly when decreasing the optical power. At the lowest fluence applied (*P*_opt_ = 2.4 mW∙cm^−2^), the photocharging time even exceeds the pulse duration within the second sequence (Figure 6f). The physics of the photocharging dynamics are based on a drift-diffusion process limited by the carrier mobility. Hence, our data (Figure 6) indicate that the effective carrier mobility strongly depends on the photocarrier concentration, which is inversely proportional to the optical power. Such a situation has been reported for different organic blends [33–35]. In some models, traps are the source of this concentration-dependent mobility of the photocarriers [36].

**Figure 7:**
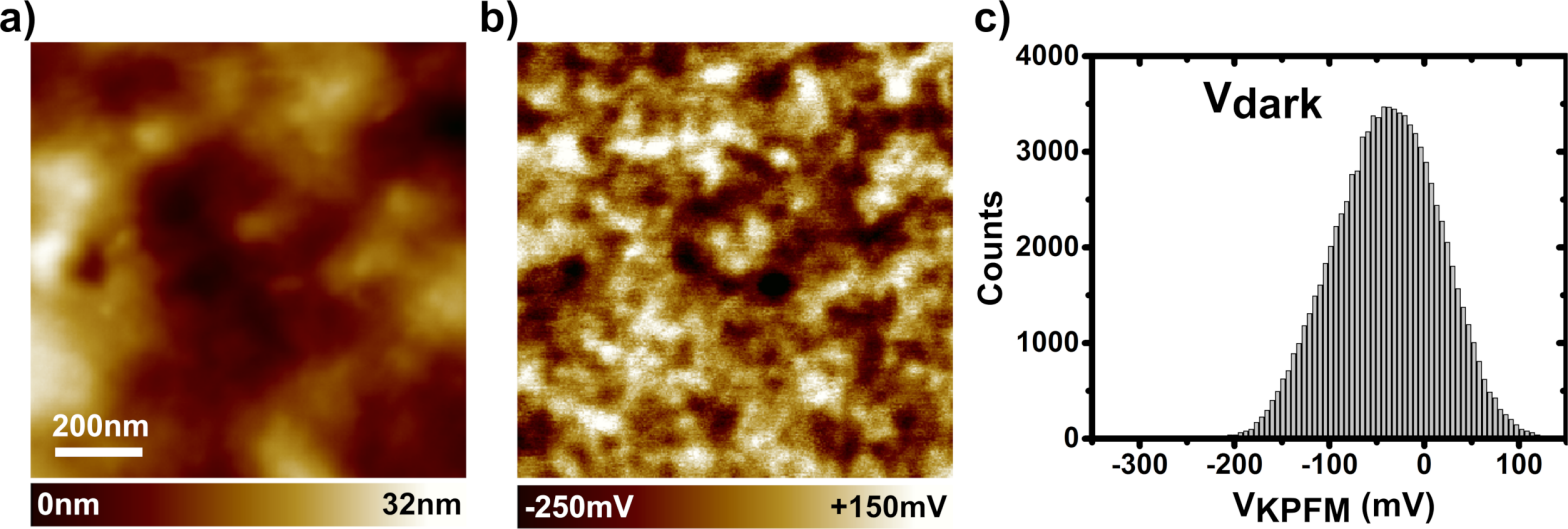
(a) Topographic (nc-AFM, UHV, 300 K) and (b) KPFM compensation potential images (1 μm × 1 μm, 300 × 300 pixels) of the PTB7:PC_71_BM blend recorded in the dark (standard KPFM configuration). (c) Histogram of the KPFM potential values.

We now focus on the photoresponse of the phase-segregated organic blend, i.e., the photoactive layer of the device without the cathode electrode, given by configuration 2 in Figure 3b. The surface morphology of the PTB7:PC_71_BM blend (Figure 7a) is consistent with that reported for samples processed under similar conditions [37–38].

The nc-AFM topographic images (Figure 7a) display a rather uniform contrast indicating that the donor and acceptor species have been finely mixed. However, specific contrasts in the KPFM potential images recorded in the dark (Figure 7b) reveal compositional and/or morphological variations at the 100 nm scale. In BHJ blends, the dark-state electrostatic contrast reflects permanent charge distributions [20], which originate from unevenly distributed electrostatic dipoles at the D−A interfaces [39] or from permanently trapped charge carriers. Obviously, if the blend composition or the morphology of the donor and acceptor sub-networks vary, the interface dipoles fluctuate as well (in direction and magnitude).

**Figure 8:**
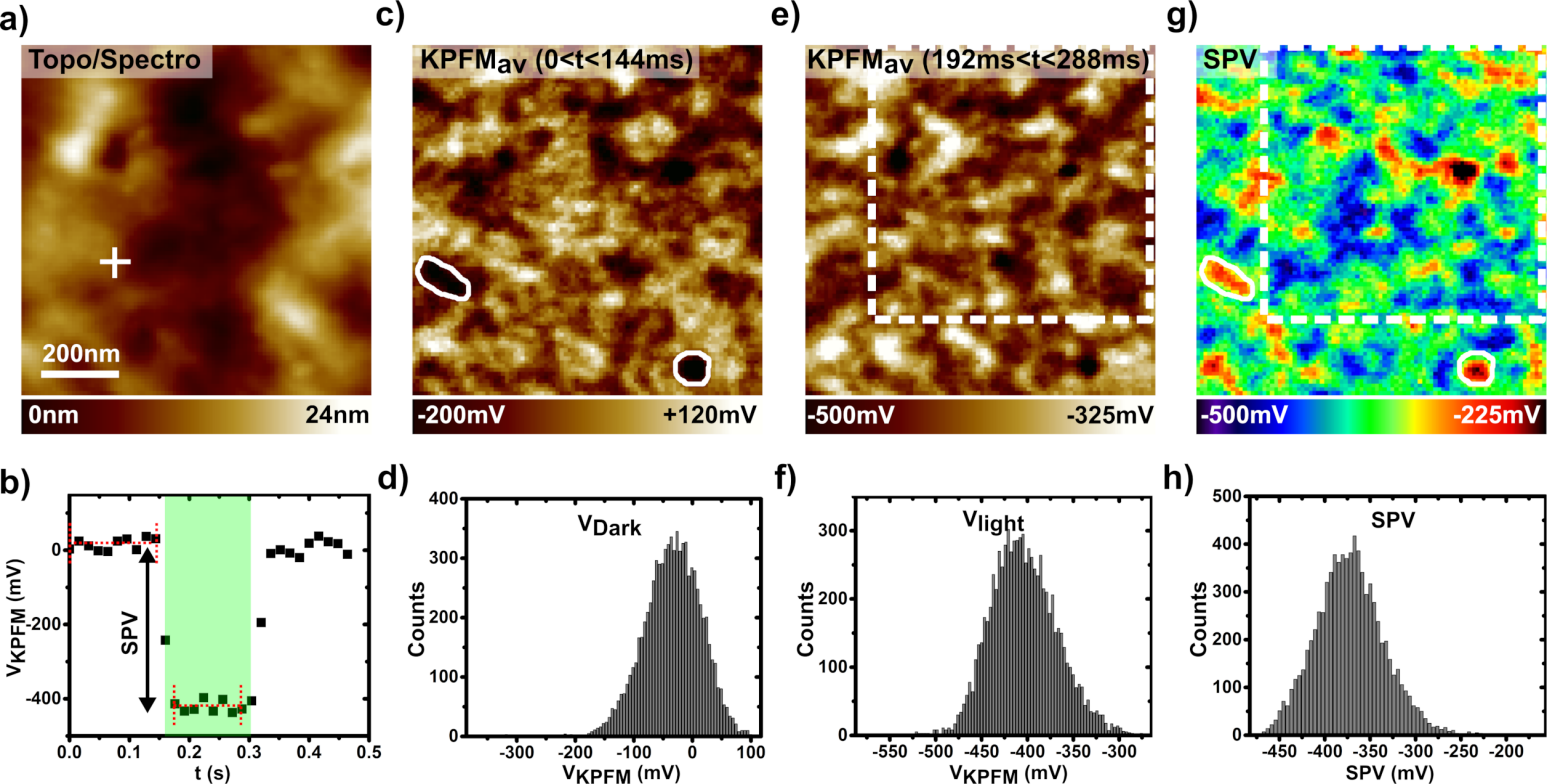
(a, c, e, g) nc-AFM/KPFM images of the PTB7:PC_71_BM blend (1 μm × 1 μm, 90 × 90 pixels) acquired during a differential SPV imaging experiment (*P*_opt_ = 18.5 mW∙cm^−2^). (a) Topographic image. (b) Spectroscopic curve of the KPFM potential as a function of time (30 pixels, each 6 ms long), recorded at the location indicated by a cross in (a). The dark-state SP and SP upon cw illumination are calculated by averaging the data points for 0 < *t* < 144 ms and 192 < *t* < 288 ms, respectively. These time windows are highlighted by red dotted lines. (c) Calculated dark-state SP (*V*_dark_) image. (d) Histogram of the *V*_dark_ values. (e) Calculated image of the SP under illumination (*V*_light_). (f) Histogram of the *V*_light_ values. (g) Calculated image of the SPV. (h) Histogram of the SPV values. The white solid contours in (c) and (g) highlight the existence of strong correlations between the contrast in the dark-state potential and the SPV images. The dotted rectangles in (e) and (g) indicate the sample area highlighted in Figure 9a–c and displayed in Figure 9e–g.

Prior to pump–probe KPFM spectroscopy, the BHJ photoresponse was investigated by differential SPV imaging (Figure 8) [40]. Here, a 2D matrix of spectroscopic curves of the KPFM potential is recorded as a function of time in a standard KPFM configuration with an open *z*-loop. During the spectroscopic ramp, the cw illumination is switched on. Images of the SP in the dark and upon illumination, as well as their difference (i.e., the SPV) can be reconstructed by simple data processing. The results are presented in Figure 8. The topographic and dark-state potential images reproduce fairly well the ones observed by the standard imaging process (compare in particular the histograms in Figure 7c and Figure 8d). The SPV is in average equal to −370 mV (Figure 8h), which is less than the SPV measured on the cathode using the same optical power (−625 mV for *P*_opt_ = 18.5 mW∙cm^−2^, see Figure 5b). This difference is not surprising, other reports [13] have already shown that the SPV probed by KPFM on the bare surface of BHJs is smaller than the open-circuit voltage [20]. More remarkable is the existence of strong correlations between the contrast in the dark-state SP and SPV images (compare Figure 8c and Figure 8g). This confirms that the local phase composition (and/or morphology) varies and has a deep impact on the opto-electronic properties of the D–A blend.

**Figure 9:**
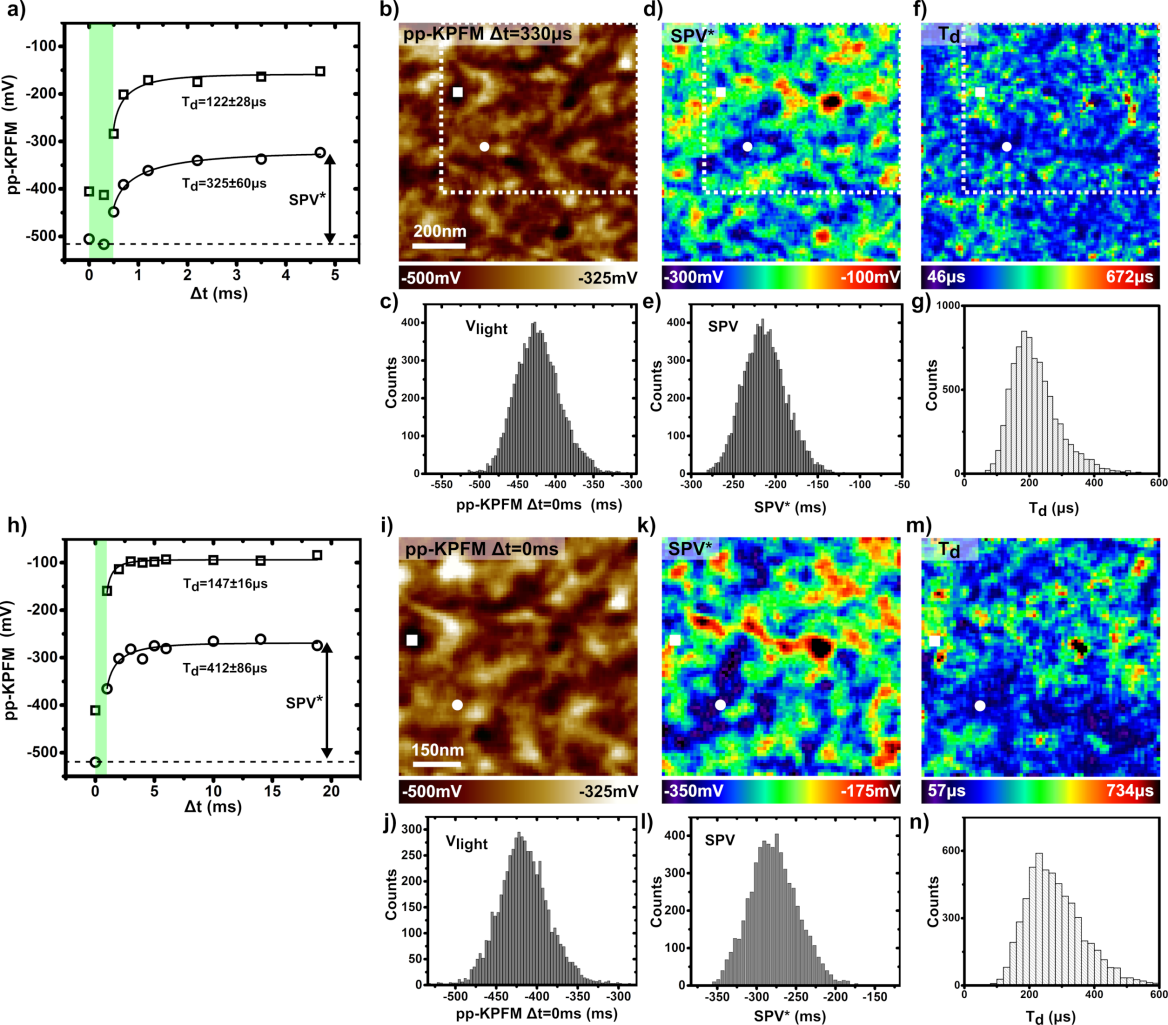
pp-KPFM spectroscopic imaging of the PTB7:PC_71_BM blend in data-cube mode. *P*_opt_ = 18.5 mW∙cm^−2^ (pulse peak power). (a–g) Data were acquired on a 90 × 90 grid using a first pump–probe sequence with pump pulses of 500 µs repeated at 200 Hz, a probe-time window of 200 µs, a data acquisition delay of 2 s and an integration time of 1 s. (h–n) Data were acquired on a 75 × 75 grid using the second pump–probe sequence with pump pulses of 1 ms repeated at 50 Hz, a probe-time window of 1 ms, a data acquisition delay of 2 s and an integration time of 1 s. (a, h) Spectroscopic curves acquired applying the first (a) and the second (h) sequence at two distinct points on the surface, which are highlighted by a circle and a square in the images. (b, d, f) Reconstructed images (first sequence) (b) of the pp-KPFM potential for a delay of 330 µs, (d) of the pseudo photovoltage SPV* (see text) and (f) of the SPV decay-time constant. (c, e, g) Corresponding histograms. (i, k, m) Reconstructed images (second sequence) (i) of the pp-KPFM potential for a delay of 0 ms, (k) of the pseudo photovoltage SPV* and (m) of the SPV decay-time constant. (j, l, n) Corresponding histograms. To reduce the fit error, all curves were adjusted by using a fixed stretch exponent β of 0.5.

Time-resolved pp-KPFM measurements in data-cube mode were performed at roughly the same sample area using two different pump–probe sequences. Spectroscopic pp-KPFM curves and 2D images recalculated from these are presented in Figure 9. To facilitate a comparison to the differential SPV images, dashed contours indicating the same sample area have been drawn in Figure 8 and Figure 9b, Figure 9d and Figure 9f. These contours correspond also to the scan area shown in Figure 9i, Figure 9k and Figure 9m.

Compared to the single-point spectroscopy measurements, 2D pp-KPFM spectroscopic imaging presents an additional degree of difficulty, especially in terms of acquisition time. To maintain a reasonable *S/N* ratio, higher probe-to-pump duty ratios were used (4% and 5% for the first and the second sequence, respectively). The pump–probe delays were set to focus on the SPV decay regime. Note that even under these conditions, several tens of hours were needed to acquire each set of images. The data shown in Figure 9a–g were acquired with pump pulses of 500 µs (*P*_opt_ = 18.5 mW∙cm^−2^) repeated at 200 Hz and a probe-time window of 200 µs. Figure 9a shows two spectroscopic curves acquired with this sequence at different points on the surface highlighted by a square and a circle in the related spectroscopic images.

Figure 9b displays the spectroscopic image of the compensation potential for a delay Δ*t* = 300 µs, which falls within the light pulse. The contrast and the potential levels (histogram in Figure 9c) are in excellent agreement with the ones obtained by differential SPV imaging under cw illumination (compare Figure 8e, Figure 8f and Figure 9b, Figure 9c). This confirms that the photocharging time is smaller than the pulse duration, in line with the results of point spectroscopy on the cathode. In a second step, one can also map a “pseudo surface photovoltage” (SPV*) image by calculating the difference between the signals measured for Δ*t* = 300 and 4.7 ms. It turns out that the SPV* (Figure 9d) and the SPV images (Figure 8g) display a similar contrast. The SPV* image however has a smaller magnitude (in average −214 mV instead of −370 mV). According to single-point spectroscopy, the potential cannot fully return to its dark-state level within a pump period of 5 ms. However, we also note that the decay-time constants obtained from the curves recorded at the bare layer are reduced by one order of magnitude compared the values acquired at the cathode. The 2D map of the SPV decay-time constants is presented in Figure 9f, and the corresponding histogram is shown in Figure 9g. At this stage, the existence of correlations between the dynamical contrasts and the ones in the dark-state SP and SPV channels seems rather unclear.

The pp-KPFM spectroscopic mapping using a longer pump periodicity yields further insights (Figure 9). The contrast of the potentiometric images (Figure 9i and Figure 9k) matches perfectly the one obtained before. Furthermore, the values of the SPV decay-time constant are consistent, although slightly higher, with the ones deduced from the measurement with the shorter pump period. A correspondence can be established between the contrast in both dynamical images. A closer examination reveals that the areas where the SPV decays the fastest correspond mainly to the ones were the SPV is more negative. The rather slow SPV decay is similar to the lifetime of long-lived trap populations reported for other BHJs [41]. The measurements on the cathode already indicated that the SPV decays reflect the trap-release dynamics. Thus, it is likely that the compositional and/or morphological heterogeneities generating the dark-state SP and SPV contrast play also a key role in the photocarrier trapping process. A reasonable (yet to be definitely confirmed) scenario could invoke the existence of non-percolating PC_71_BM clusters acting as trapping centers. Such an imperfect morphology would be consistent with the relatively low macroscopic performance of this device compared to established PTB7:PC_71_BM devices (Figure S3, Supporting Information File 1).

Finally, some questions remain open and will require further investigations. Firstly, the origin of the difference between the SPV decay-time constants determined in the bare layer and the cathode measurements remains unclear. To address this point, statistical measurements at several sample areas would be needed to check if the sample properties are homogeneous or if they display strong variations at the mesoscopic scale. Indeed, sample parts with longer trap-release times may exist, which would contribute to the global SPV decay dynamics probed on the cathode. Secondly, the difference between the SPV magnitudes probed by differential and pp-KPFM 2D imaging is still unexplained. This difference decreases significantly when increasing the pump period (see the histograms in Figure 9). However, even at the longest pump period, the dark-state level is not recovered completely. This is puzzling since such an effect was neither observed for the point-spectroscopy measurements on the cathode, nor for the pp-KPFM measurements using electrical pumping. A comparison of the differences of these three experimental configurations may yield further insights. Optical pumping of the bare organic layers presents a specificity. Both the tip–sample capacitance and the KPFM *S*/*N* ratio are susceptible to significant changes with varying illumination state. Upon illumination, the charge carrier density increases leading not only to a reduction of the tip–sample capacitive junction but also to an increase in the KPFM *S*/*N* ratio. Regarding the basic operating principles of pp-KPFM, it is unclear why photoinduced capacitive changes shall affect the measurement of the dark-state potential only. An illumination-dependent *S*/*N* ratio may be a better explanation similar to previous observations by Murawski et al., who have shown [22] that the electrostatic contrast cannot be fully measured for very small electrical pumping cycles. Here, the *S*/*N* ratio becomes too low to perform stable pp-KPFM experiments at a useful bandwidth. To verify this hypothesis, further experiments and developments are needed. In particular, we plan to increase the *S*/*N* ratio by using a heterodyne scheme [6] instead of the standard frequency-modulation mode for the KPFM operation.

## Conclusion

We have introduced an alternative approach to pp-KPFM based on the acquisition of spectroscopic curves of the KPFM compensation potential as a function of the pump–probe delay using an open-loop configuration. This configuration simplifies the operation of pp-KPFM, since it allows for avoiding topographic artefacts without the need to use a second compensation loop. Single-point spectroscopy measurements performed on HOPG (under electrical pumping) and on an organic solar cell cathode (under optical pumping), confirmed the validity of this implementation. In addition, we demonstrated that spectroscopic pp-KPFM can be used in data-cube mode enabling the acquisition of 2D images of the SPV dynamics of organic BHJs. In the investigated PTB7:PC_71_BM blend, the SPV decay dynamics were found to be dominated by trap-release processes at the time scale of a few hundreds of µs to a few ms. The resolution of this pp-KPFM approach is however not limited to these slow dynamics. A temporal resolution as good as 1 µs was already obtained using electrical pumping, and further developments are in progress to perform ns-resolved measurements. This will be done by implementing a multiplication stage generating the probe signal and by modifying the electric circuit for fast operation upon electrical pumping with a correct circuit impedance matching.

Last, by comparing the results of pp-KPFM and of differential SPV imaging performed on the bare organic layer, an underestimation effect in the measurement of the SPV has been evidenced. These results stress the need to quantify properly the dependence of the *S*/*N* ratio of the pp-KPFM potential on the illumination state when investigating photovoltaic materials. For that purpose, future works will be devoted to increasing the sensitivity of spectroscopic pp-KPFM, for instance by using a heterodyne or a side-band detector. Further insights will also be gained by comparing different kinds of photovoltaic materials, for which the illumination-dependence of the *S*/*N* ratio may be different. In particular, we plan to investigate silicon-based devices, hybrid perovskite thin films and single crystals as well as type-II van der Waals heterojunctions based on transition metal dichalcogenides.

## Experimental

### Nc-AFM and pp-KPFM

Noncontact-AFM (nc-AFM) experiments were performed with a ScientaOmicron VT-AFM setup in UHV at room temperature (RT) driven by a Matrix SPM control unit. Pt/Ir-coated silicon cantilevers (EFM, Nanosensors, resonance frequency in the 45–115 kHz range) were annealed in situ to remove atmospheric contaminants. Topographic imaging was realized in FM mode (FM-AFM) with negative frequency shifts of a few Hz and vibrational amplitudes of a few tens of nm. KPFM measurements were carried out in single-pass mode using FM (FM-KPFM) with a peak-to-peak modulation bias *V*_ac_ of 0.9 V at 1140 Hz. The compensation voltage *V*_dc_ was applied to the cantilever (tip bias *V*_tip_ = *V*_dc_). The CPD is therefore the negative of *V*_dc_, hence *V*_tip_ = *V*_dc_ = −CPD [39]. The KPFM data are presented as *V*_dc_ images also referred to as KPFM potential or SP images for simplicity. A lock-in amplifier (Signal Recovery 7280) was used to measure simultaneously the modulation of the frequency shift at the electrostatic excitation frequency. The ‘in-phase’ amplitude of the first harmonic is fed into the KPFM bias feedback loop of the SPM controller. A fast radiofrequency analog switch (ZASWA-2-50DRA+, Mini-circuits, switching time rated to 20 ns) was used to apply a “pseudo multiplication” on the sum of the KPFM compensation bias and the ac bias generated by the LIA output. The switch driver TTL input was connected to the first output channel of a programmable AWG (Keysight 33622A). That channel was dedicated to the generation of the probe signal. Note that in the “open state” the switch output is grounded.

Both probe and pump signals were generated by a programmable dual channel AWG (Keysight 33622A), which was synchronized with the scanning probe microscopy (SPM) unit by its external trigger input. The pulse sequences were programmed using the Keysight BenchLink Waveform Builder Pro software. Basically, the pulses consisted in a series of dual waveforms, each of them featuring a different delay between the two channels, which were repeated until a trigger event was sent to the AWG. The two channels are initially synchronized after downloading the sequence to the unit by using the “sync arbs” function of the 33622A unit.

In optical pumping configuration, the second channel of the AWG was used to drive the digital-modulation input of a fiber-coupled PhoxXplus laser module operated at 515 nm (Omicron Laserage GmbH). Sample illumination was performed in backside geometry using custom sample holders with on-board mirrors through an optical viewport of the UHV AFM chamber. The wavelength of 515 nm was selected since it falls within the UV–vis absorption bands of both PC_71_BM and PTB7. For each measurement, the optical power *P*_opt_ corresponding to the maximum pulse intensity during the modulated illumination is indicated in the corresponding figure caption (Figure 5, Figure 8, Figure 9). *P*_opt_ is defined per unit of surface by taking into account the laser beam diameter.

### Image processing and processing of the spectroscopic data

The WsXM software [42] was used to process the SPM images. Semi-automated data processing routines were also developed using the batch processing options of the OriginPro software (OriginLab Corp.). These routines were employed to import the 2D spectroscopic data and to perform automated curve fit adjustments on the VKPFM(Δ*t*) curves. A Gaussian smooth filter was applied to the spectroscopic images. The processed data display the same features as the raw data however at a slightly lower noise level.

### Organic BHJ thin films processing, solar cell fabrication and characterization

The PTB7:PC_71_BM BHJ thin film was deposited on an indium thin oxide (ITO) substrate coated with PEDOT:PSS (a hole-conducting polymer) following the procedure published by Liang et al. [28]. PTB7 (Ossila, *M*_w_ = 85 kDa, PDI = 2.0) and PC_71_BM (Solenne BV, 99% purity) were used as received. A thin layer of filtered (0.45 µm) PEDOT:PSS (Baytron A14083, Clevios) was spin-coated onto the activated ITO surface at 5000 rpm for 25 s, 4000 rpm for 60 s and 4000 rpm for 1 s (≈30 nm) and annealed at 120 °C for 10 min under ambient conditions. The substrate was then transferred into an argon-filled glovebox for spin-coating of an active layer of the PTB7:PC_71_BM solution (1:1.5 weight ratio, 25 mg∙mL^−1^ total concentration) in anhydrous chlorobenzene. The blend was stirred overnight at 50 °C for complete dissolution and cooled down to RT. Then, 3 vol % of 1,8-diiodoctane were added 2 min before deposition at 1500 rpm for 120 s and 1000 rpm for 1 s on a substrate preheated to 50 °C (110 ± 10 nm). The sample was finally loaded into a secondary vacuum deposition system (Kurt J. Lesker) for deposition of Ca (20 nm, 1.0 Å∙s^−1^) and Al (100 nm, 2.0 Å∙s^−1^) top electrodes (10.18 ± 0.1 mm^2^). The electrical characterization was performed in a glovebox. Current-density–voltage (*J*–*V*) curves were measured using a Keithley 2400 source measure unit. The photocurrent was measured under AM 1.5G illumination at 1000 W∙m^−2^ using a Newport Thermal Oriel 91192 1000W solar simulator. The light intensity was calibrated using a monocrystalline silicon Oriel Newport 91150v VLSI reference solar cell certified by the National Renewable Energy Laboratory (NREL).

## Supporting Information

File 1 Further experimental measurements, details of the pp-KPFM experiment, characterization of the solar cell device and derivation of the formula used to fit the pp-KPFM spectroscopy.
