# Supplementary material for: Implementation of data-cube pump–probe KPFM on organic solar cells
Source: Beilstein J Nanotechnol. 2020 Feb 12;11:323–37. doi: 10.3762/bjnano.11.24 (PMC7034223; doi:10.3762/bjnano.11.24)
Supplement: File 1 — Further experimental measurements, details of the pp-KPFM experiment, characterization of the solar cell device and derivation of the formula used to fit the pp-KPFM spectroscopy. [file Beilstein_J_Nanotechnol-11-323-s001.pdf]

BEILSTEIN JOURNAL OF NANOTECHNOLOGY

Supporting Information

for

Implementation of data-cube pump–probe KPFM on organic solar cells

Benjamin Grévin, Olivier Bardagot and Renaud Demadrille

Beilstein J. Nanotechnol. **2020,** *11,* 323–337. doi:10.3762/bjnano.11.24

Further experimental measurements, details of the pp-KPFM experiment, characterization of the solar cell device and derivation of the formula used to fit the pp-KPFM spectroscopy

License and Terms: This is a supporting information file under the terms of the Creative Commons Attribution License (https://creativecommons.org/licenses/by/4.0). Please note that the reuse, redistribution and reproduction in particular requires that the authors and source are credited.
The license is subject to the *Beilstein Journal of Nanotechnology* terms and conditions: (https://www.beilstein-journals.org/bjnano)

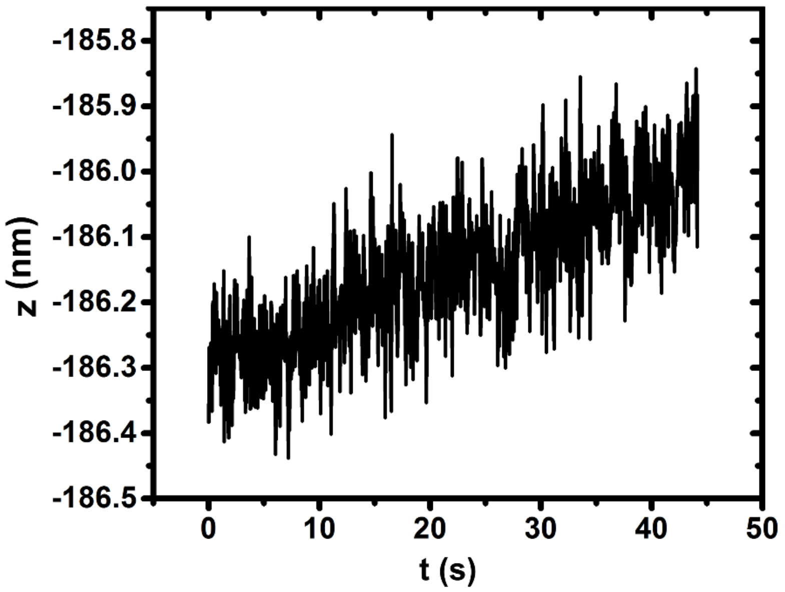

Figure S1: Plot of the *z*-position (*xy*-scan stopped) as a function of time. The *z*-drift is smaller than 0.4 nm over 40 s.

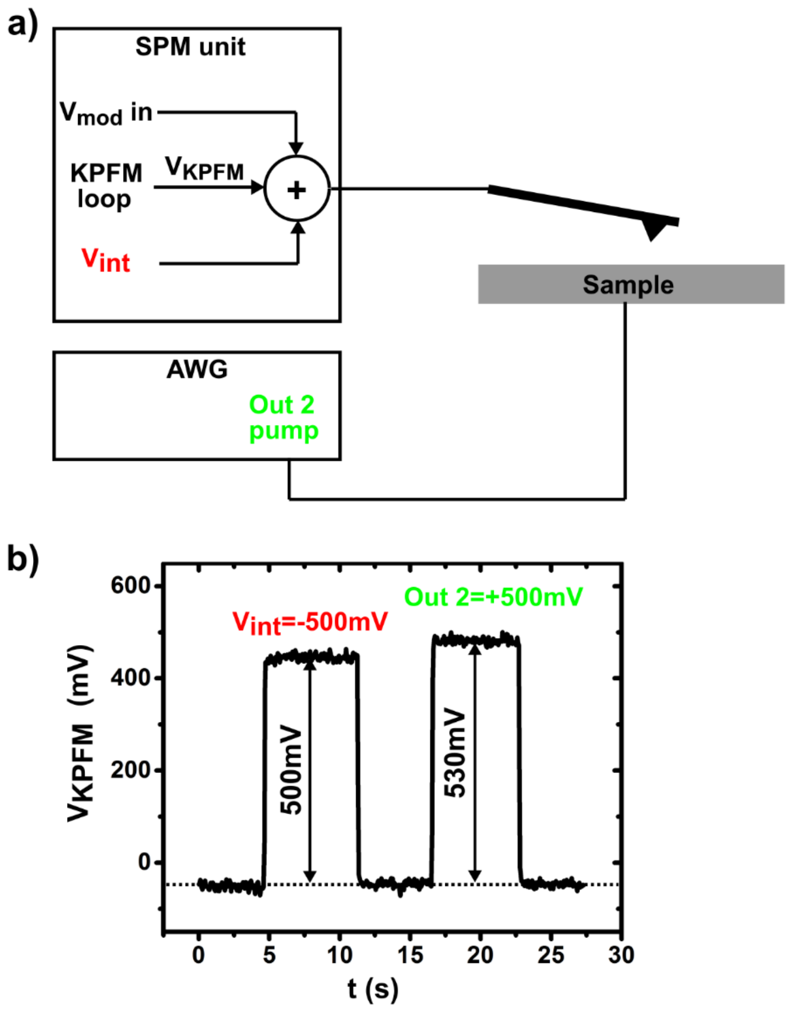

Figure S2: (a) Simplified scheme of the cantilever tip and the electrical connections. An additional bias voltage (V_{int}) can be added to the tip by the SPM unit. The sample is connected to the second output (pump channel) of the AWG. (b) Plot of the KPFM compensation potential as a function of time. The KPFM potential increases exactly by +500 mV when an internal DC bias of –500 mV is added to the tip. When a +500 mV signal is applied to the sample by the AWG, the compensation potential is shifted by +530 mV. We attribute this difference to an effect of impedance mismatch in the electrical connections. Note that the overestimation factor (530/500 = 1.06) is identical to the one observed when measuring a square wave pulse by pp-KPFM under electrical pumping (with a measured amplitude of 213 mV instead of 200 mV).

a)

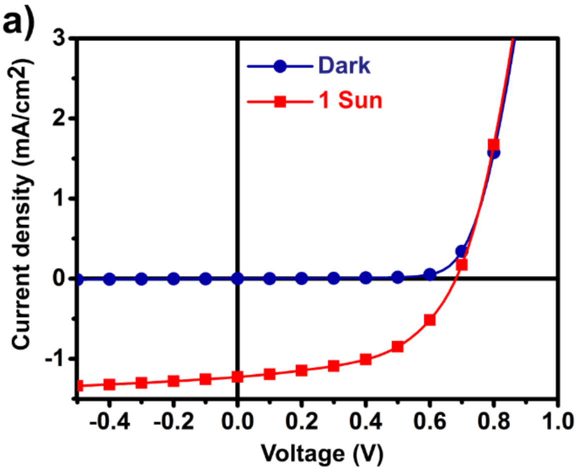

b)

System	Architecture	Ratio	Solvent (vol %)	Area (mm^2)	J_{SC} (mA/cm^2)	V_{OC} (V)	FF(%)	PCE(%)	Ref
PTB7:$PC_{71}BM$	Conventional	(1:1.5)	CB:DIO (97:3)	10.18	-12.02	0.68	51	4.20	This work
PTB7:$PC_{71}BM$	Conventional	(1:1.5)	CB:DIO (97:3)	10.00	-14.50	0.74	69	7.40	Liang *et al.*

Figure S3: (a) Current–voltage curves of the solar cell device in dark state and under illumination (AM 1.5G, 1 sun). (b) Main performance parameters. J_{sc}: short circuit current. V_{oc}: open circuit voltage. FF: fill factor. PCE: power conversion efficiency. First line: our work, second line: data after Liang, Y.; Xu, Z.; Xia, J.; Tsai, S.-T.; Wu, Y.; Li, G.; Ray, C.; Yu, L. *Adv. Mater. (Weinheim, Ger,)* **2010,** *22,* E135–E138. doi:10.1002/adma.200903528

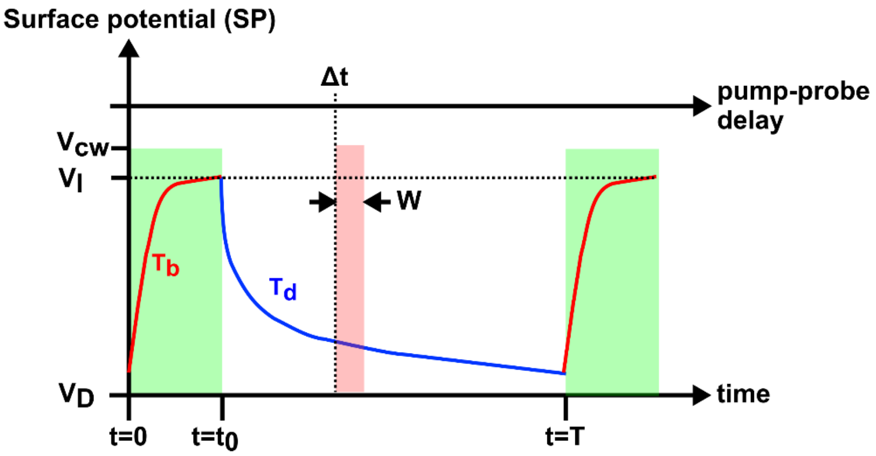

Figure S4: Time-evolution of the surface potential (SP) under pulsed illumination. The time-intervals corresponding to the pump and probe signals (shown only for one given delay, Δt) are highlighted by half-transparent green and red rectangles. The illumination occurs for $0 \leq t \leq t_0$. W: probe time-window. T: pump signal period. V_D: surface potential in dark. V_I: maximum value of the surface potential under illumination. If the pulse duration exceeds largely the photovoltage build-up time constant (τ_b), V_I equals the SP value that would be measured under continuous wave illumination (V_{cw}). τ_d: photovoltage decay time constant.

In the following, we derive the formula used to fit the decay part ($t_0 \leq \Delta t$) of the pp-KPFM spectroscopic curves. The surface photovoltage (SPV) decay dynamics are considered by using a stretched exponential function (with time constant τ_d and stretch exponent β):

for $t_0 \leq t \leq T$, $SP(t) = (V_\mathrm{I} - V_\mathrm{D}) \times e^{-[(t-t_0)/t_\mathrm{d}]^{\beta}} + V_\mathrm{D}$ (1).

The potential probed by pp-KPFM corresponds to the time-averaged value of *SP*(*t*) over the probe time window:

$\gamma(s,x) = \int_0^x t^{(s-1)} \times e^{-t} dt$, with $t_\mathrm{i} = \Delta t$ (2).

Below we calculate the time-averaged value of the exponential term in (1). The potential probed by pp-KPFM corresponds to the time-averaged value of SP(t) over the probe time window:

$$\frac{1}{w}\int_{t_\mathrm{i}}^{t_\mathrm{i}+w} e^{-[(t-t_0)/t_\mathrm{d}]^{\beta}} dt = \frac{t_\mathrm{d}}{w \times \beta} \times \left(\gamma\left[\frac{1}{\beta}, \left(\frac{t_\mathrm{i}+w-t_0}{t_\mathrm{d}}\right)^{\beta}\right] - \gamma\left[\frac{1}{\beta}, \left(\frac{t_\mathrm{i}-t_0}{t_\mathrm{d}}\right)^{\beta}\right] \right) \quad (3)$$

with $\gamma(s,x) = \int_0^x t^{(s-1)} \times e^{-t} dt$ (not normalized lower half Euler Gamma function)

Thus, for $t_0 \leq \Delta t$:

$$pp-KPFM(\Delta t) = (V_\mathrm{I} - V_\mathrm{D}) \times \frac{t_\mathrm{d}}{w \times \beta} \times \left(\gamma\left[\frac{1}{\beta}, \left(\frac{\Delta t+w-t_0}{t_\mathrm{d}}\right)^{\beta}\right] - \gamma\left[\frac{1}{\beta}, \left(\frac{\Delta t-t_0}{t_\mathrm{d}}\right)^{\beta}\right] \right) + V_D \quad (4)$$

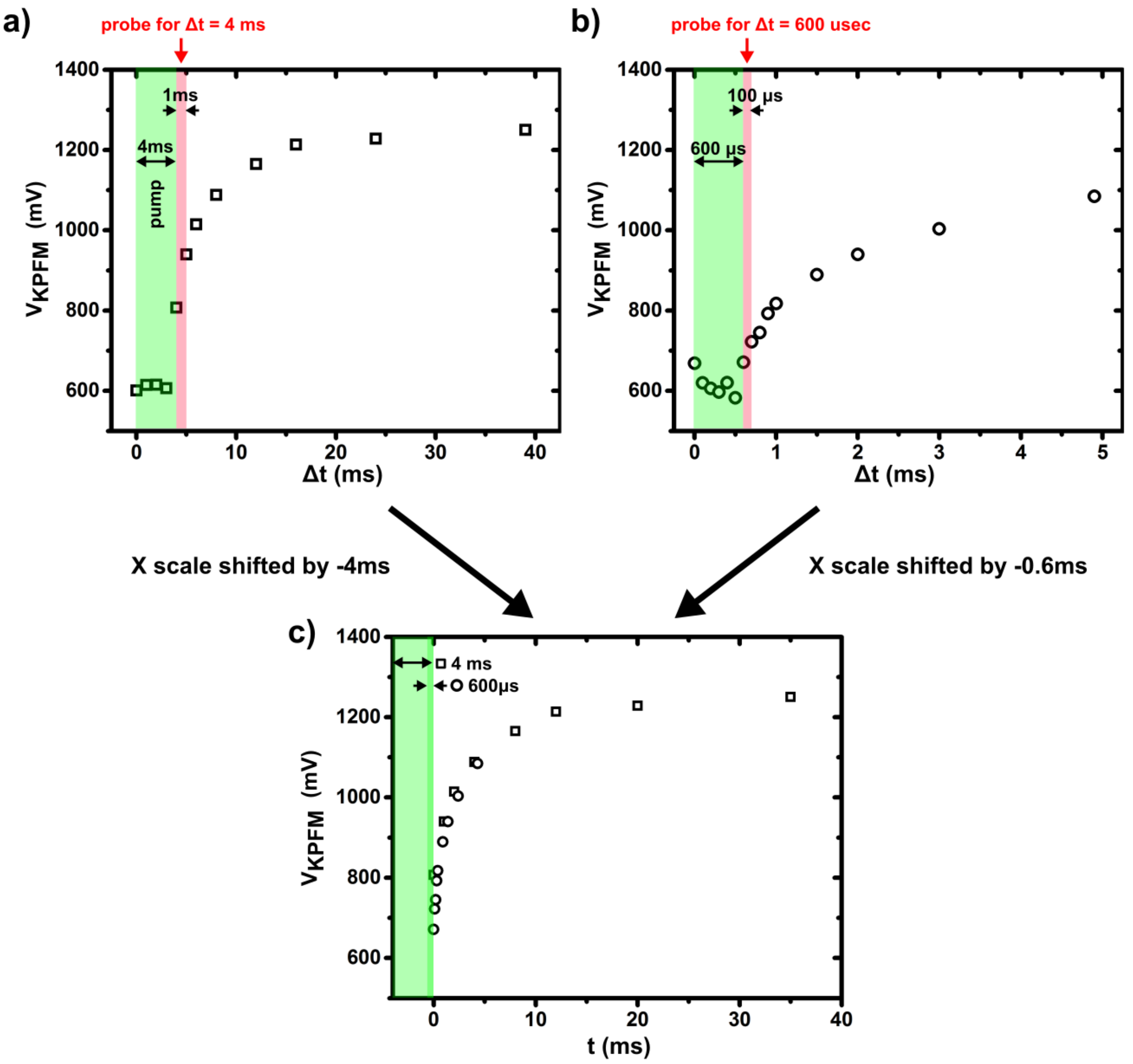

Figure S5: Consistency check concerning two data sets acquired for different pump-probe sequences (point spectroscopy on the solar cell cathode, P_{opt} = 193 mW·cm^{-2}, the same data are presented in Figure 6a,b). (a) First sequence: 4 ms pump pulses repeated at 25 Hz, 1 ms probe time window. (b) Second sequence: 600 µs pump pulses repeated at 200 Hz, 100 µs probe time window. (c) To merge the two curves in the same plot, the time origins of the spectra are shifted so that t = 0 s for both corresponds to the light pulse extinction.

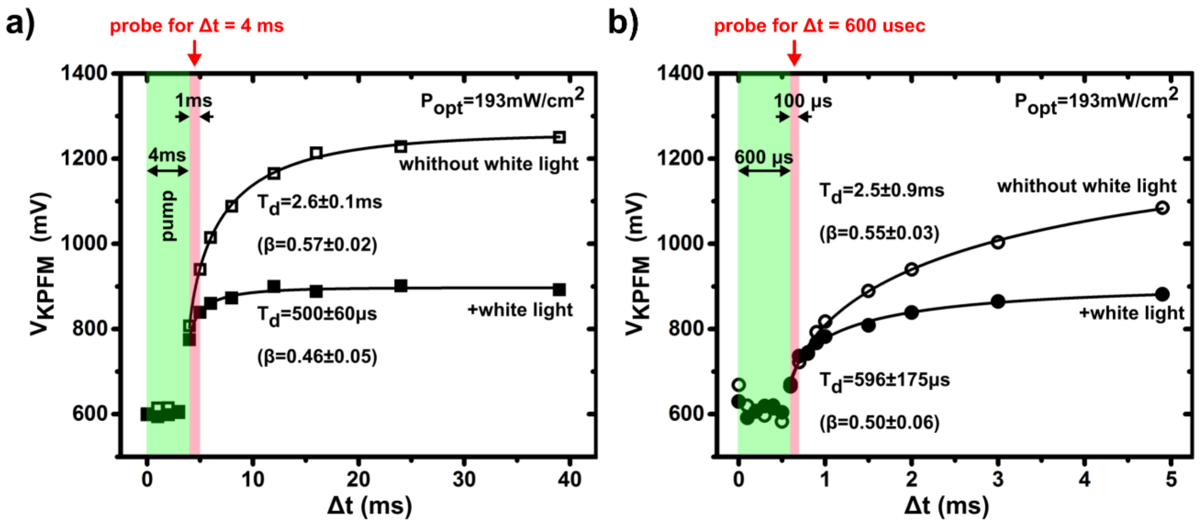

Figure S6: (a,b) Spectroscopic curves (averaged from 5 acquisitions) of the pp-KPFM compensation potential as a function of the pump probe delay (Δt) measured at the solar cell cathode. The data plotted with open symbols are also presented in Figure 6a,b. The data plotted as filled symbols have been acquired using the same experimental parameters plus a white light background of fixed intensity. This was realized by turning on the lamp used to illuminate the sample stage (otherwise kept in dark by cover-windows). The optical power of this "pseudo" white light source was not accurately calibrated, and is estimated to be at maximum on the order of a few hundreds microwatts per square centimeter. The solid lines represent the results of the numerical fits performed to extract the SPV decay-time constants (τ_d) and the stretch exponents (β).
